# Full-scale experimental investigation of deposition and corrosion of pre-protector and 3^rd^ superheater in a waste incineration plant

**DOI:** 10.1038/s41598-017-17438-3

**Published:** 2017-12-13

**Authors:** Wenchao Ma, Terrence Wenga, Nan Zhang, Guanyi Chen, Beibei Yan, Zhihua Zhou, Xiao Wu

**Affiliations:** 1 Tianjin Engineering Center of Biomass-derived gas/oil Technology/State Key Laboratory of Engine, School of Environmental Science and Engineering, Tianjin University, Tianjin, 300072 China; 2 China Construction Engineering Design Group Corporation Limited (Headquarter), Beijing, 100037 China; 3 School of Science, Tibet University, Lhasa, 850012 China; 4 China National Environmental Protection Corporation, Beijing, 100082 China

## Abstract

Municipal solid waste (MSW) incineration is widely adopted as a waste management strategy and for the energy production. However, this technology experience grave deposition and corrosion of the boiler tubes due to high chlorine (~1.09wt.%) and alkali metal (Na, K) content in MSW. Little is known about the concentration profile of these corrosive elements in the deposits at different boiler locations. Therefore, a full-scale experimental investigation was conducted to determine the concentration profile of Cl, K, Na, S, and Ca in the deposits at pre-protector and compare with those at 3^rd^ superheater during MSW combustion at a 36 MWe waste incineration plant (WIP) in Chengdu, China. The deposit samples were analyzed using wet chemical techniques, scanning electron microscope coupled with energy dispersive spectroscopy (SEM/EDS), and X-ray diffraction (XRD). The concentrations of Na, K, and Cl were high in the deposits at pre-protector while S and Ca concentrations were high on the 3^rd^ superheater. The pre-protector was severely corroded than the 3^rd^ superheater. The governing mechanisms for the deposition and corrosion on these boiler locations were elucidated.

## Introduction

Along with the rapid population growth and industrialization, the energy demands are constantly rising, leading to the increase in fossil fuel consumption. China, one of the greatest fossil fuel consumer in the world, utilized approximately 2.7 Gt of coal in 2007 while in 2013 approximately 4.3 Gt. This immense consumption had led to the decline of fossil fuel reserves^1^ and had contributed much to the incomparable environmental problems such as severe smog in China^2^. Considering these problems, combustion of municipal solid waste (MSW) to generate electricity had gained much attention^3,4^ and can reduce the net CO_2_ emission per heating value of coal and natural gas by 93% and 84% respectively^5^. The capacity of MSW incineration in China increased from 3.7 million tons in 2003 (2.5%) to 23.2 million tons in 2010 (14.7%)^6^. In 2014, there were 188 waste incineration plants (WIP) in China, running with a total combustion capacity of 1.86 × 10^5^ tons per day (32.5%), producing 3.72 GWh electricity per day^7,8^ and the biomass power installed capacity is expected to reach 30 GW of electricity, accounting for 3% of the total installed capacity in 2020^9^.

However, combustion of MSW, which has a high concentration of Cl (0.45–0.72wt.%)^10^, (0.5–1.00wt %)^11^ and alkali metals (K and Na) result in severe ash deposition and corrosion of the boiler tubes^11–16^. It is estimated that deposition and corrosion of the heat transfer surfaces in WIP result in reducing electricity generation by approximately 0.5–1.5%^17^.

Up to date, studies have been focused on the bottom ash, fly ash, deposits, and corrosion of the superheater only in MSW incinerators^16,18,19^ while other boiler parts received little attention. A previous study by Chen *et al*.^20^ found that the elemental composition of the deposits in different parts of the boiler varies due to the changes in flue gas temperature and hence the governing mechanisms are not the same^21^. Currently, studies have reported that flue gas from the combustion chamber has a large amount of fly ash, consisting of particles ranging from submicron to millimeter^19^. They contain alkalis which act as glue for the secondary deposition that consists of matrixes of anhydrite calcium phase (CaSO_4_), where silicates, sulfates, chlorides, and oxides are incorporated as solid particles derived from the combustion chamber^21^. These compounds undergo several physical and chemical reactions and eventually build up the deposits on the surfaces of the tubes through thermophoresis and turbulent diffusion^12^. Within the deposits, mineralogical reactions between the chlorides, sulfates and the flue gas take place. Coarse fly ash particles then adhere to the initial sticky layer on the surfaces of the boiler^19^. Moreover, heavy metal chlorides in the deposits, as low-melting compounds, lead to a decrease of the first melting temperature of the ash deposits down to 200 °C–300 °C^22^, and subsequently cause dramatic corrosion on steel surfaces due to the increased chemical reactions as well as providing an electrolyte for transport of ions and chemical attack^5^.

According to Pfrang-Stotz *et al*.^23^, the deposits from a WIP comprises largely of various sulfates of Ca, Na, K, Pb, and Zn with small contents of oxides, silicates, chlorides, and phosphates^19,24^. This mineralogical composition of the deposits changes with location in the same boiler and in different types of boilers as deposition is influenced by several factors including, feedstock, type and geometry of the boiler, flue gas velocity, and temperature^21^. Changes in the deposits composition have different influences on the boiler tube corrosion.

Although extensive research on deposition and corrosion in WIP has been published, no data have been reported on the concentration profile of Na, K, Cl, Ca, and S in the deposits at pre-protector in WIP. Ash deposition and corrosion of WIP in Chengdu, China was studied. To our knowledge, high temperature corrosion usually occurred at 3^rd^ superheater area. However, in the Chengdu waste incineration plant, the boiler actually had suffered from severe corrosion at the pre-protector. As a result, a need was identified to study the deposit chemistry on the pre-protector and compare with those on the 3^rd^ superheater which most studies had put much focus on. The objectives of this study were to determine the amount of corrosive species in the deposits of these two heat exchangers, evaluate and compare relative severity to high temperature corrosion. The research consists of investigating the surface morphology, elemental composition and concentration distribution in the deposits. The information is useful in applying optimum corrosion measures and optimization of the combustion environment thus helps in alleviating deposition and corrosion in WIP.

## Results

### Morphological SEM analysis

**Figure 1:**
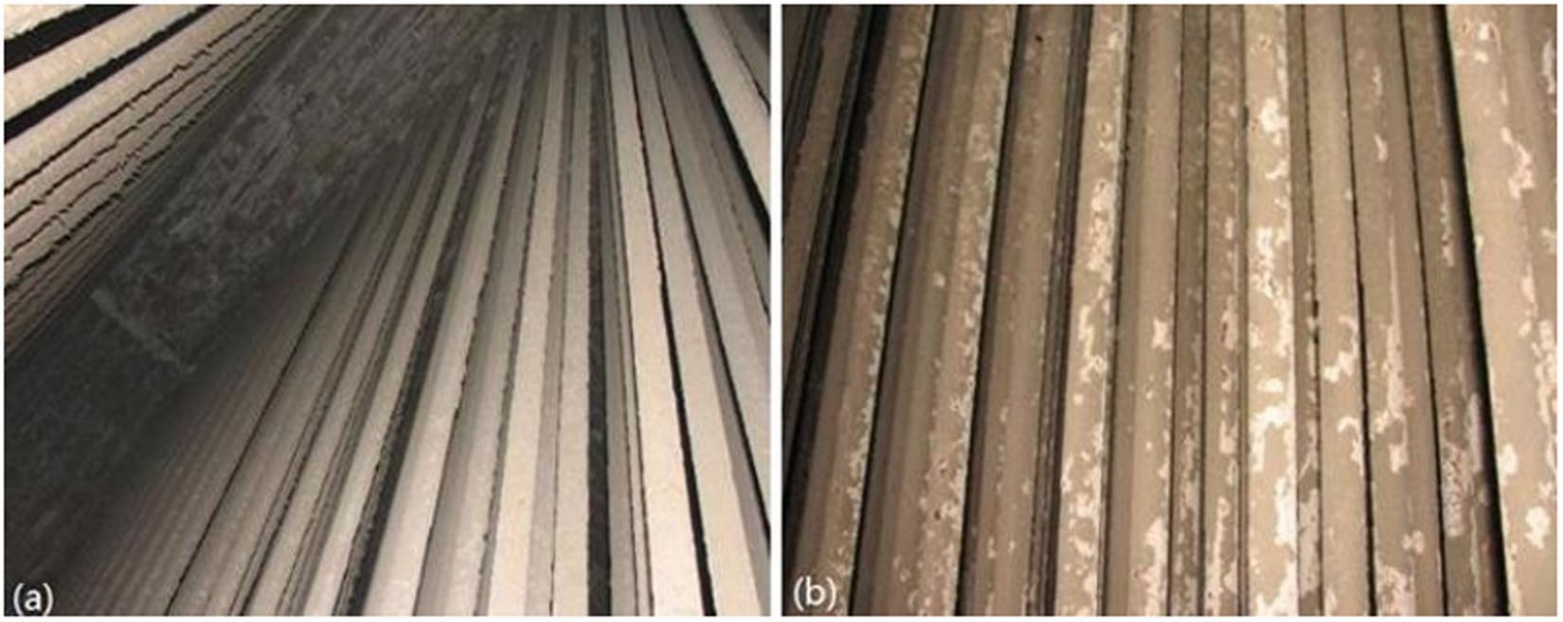
Ash deposits on (**a**) pre-protector and (**b**) 3^rd^ superheater.

Large amounts of deposits were formed on the pre-protector and the 3^rd^ superheater as shown in Fig. 1. The ash deposit layer on the pre-protector was much thicker and denser than on the 3^rd^ superheater.

**Figure 2:**
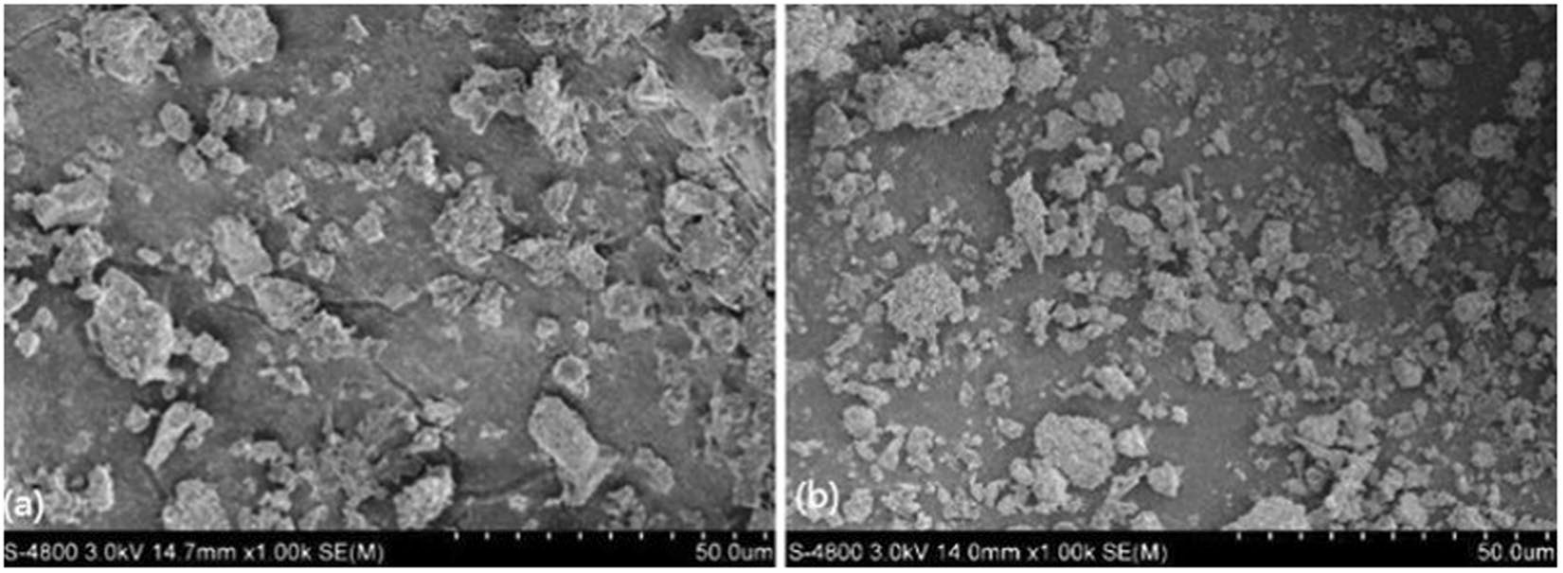
SEM images of deposits on (**a**) pre-protector and (**b**) 3^rd^ superheater.

The ash deposits on the 3^rd^ superheater, in the brownish color, were thinner and some of them peeled off from the tube surface, indicating that severe corrosion had occurred. Figure 2 shows the SEM microstructure of the ash deposits at the pre-protector and the 3^rd^ superheater. All ash deposit particles in the two pictures were in random-sizes and irregular shapes with no obvious tendency of aggregation. The particles were of different kinds of shapes including elongated, spherical, cube-like, plate-shaped and hemispherical. This observation is in line with previous studies^25,26^.

**Figure 3:**
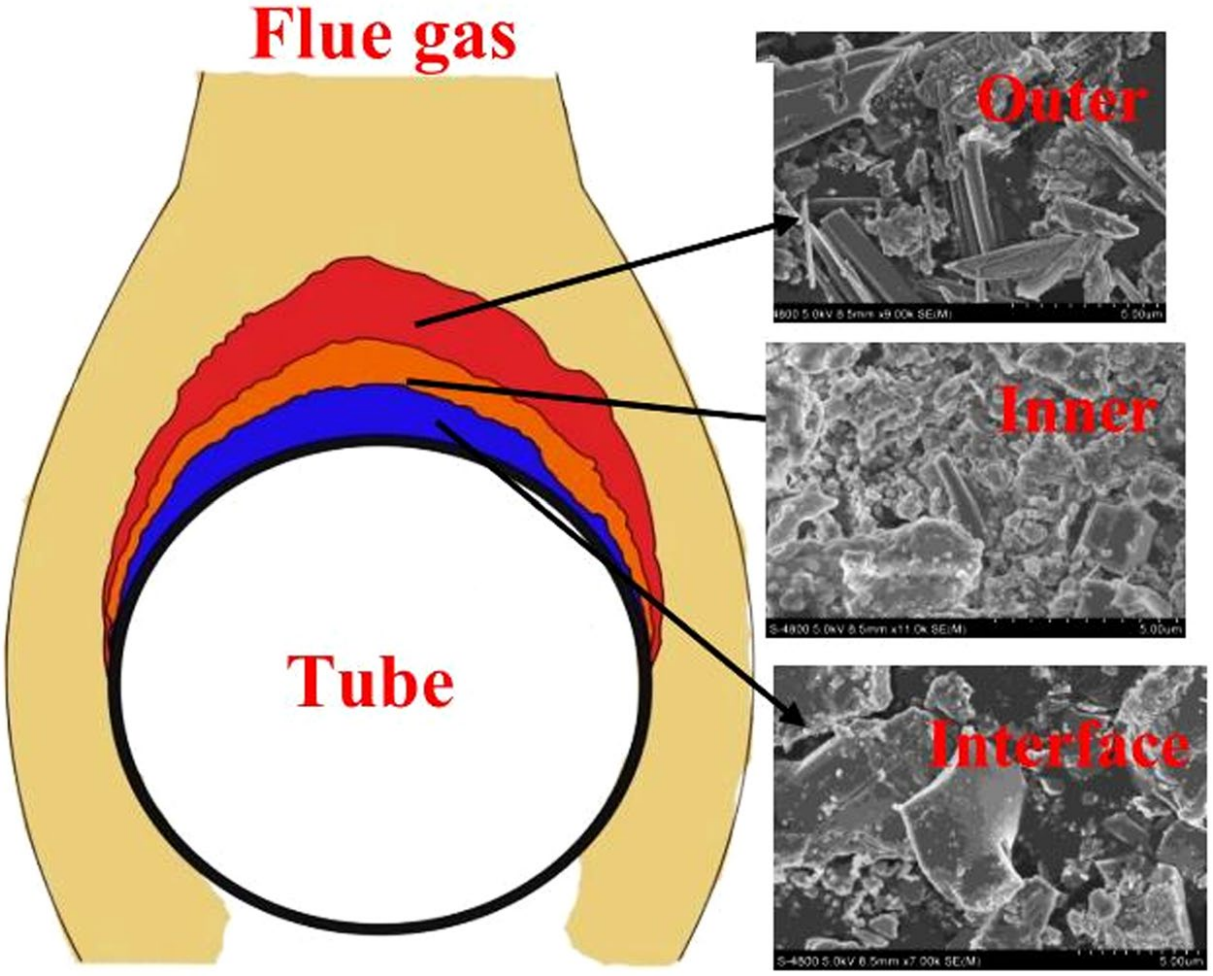
SEM micrograph of deposits on pre-protector in terms of growth.

In order to detect the morphology of the deposit particles in terms of deposit growth, the ash deposits on the pre-protector were collected and were classified into the outer, the inner and the interface layer. SEM was conducted and it was visible that three layers had different microstructures as shown in Fig. 3. The outer deposit layer had some particles in the shape of sticks, while the inner deposit layer had leaf-like shaped particles with holes accumulating together and the interface deposit layer had angular shaped and individual particles.

### Chemical analysis by EDS

**Figure 4:**
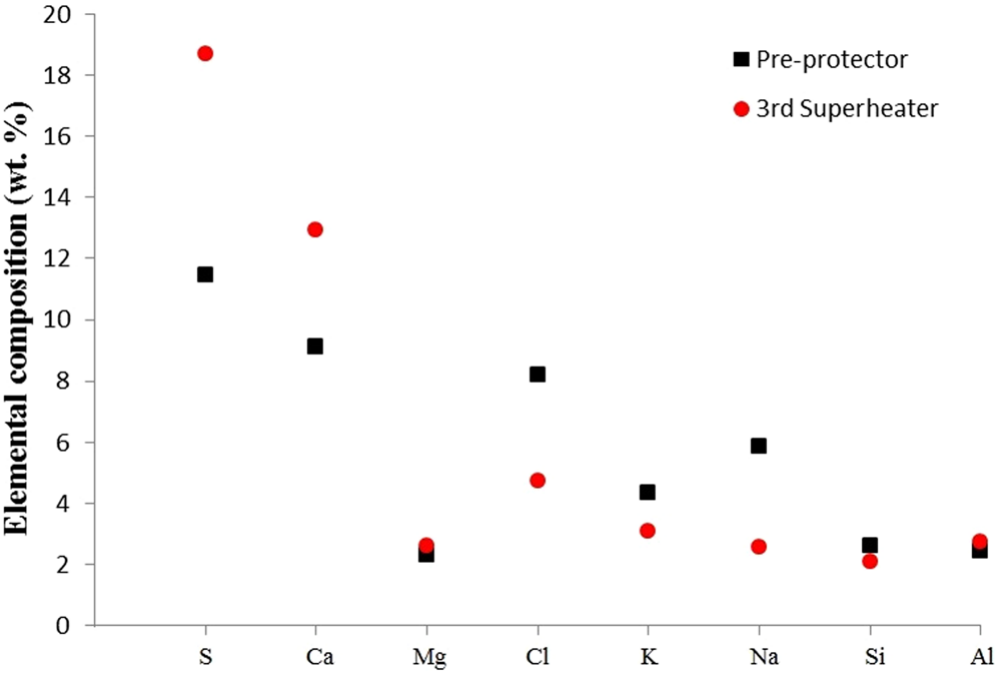
Elemental analysis of ash deposits on pre-protector and 3^rd^ superheater.

The chemical composition of the deposits from the pre-protector and the 3^rd^ superheater was determined using the EDS technique. The analysis showed that sulfur was dominant in both locations. In addition, the deposits on the 3^rd^ superheater had more Ca as the major element while the pre-protector had Na, K, and Cl as the core elements (see Fig. 4). Magnesium, silicon, and aluminum were detected in small amounts at both locations.

### Wet chemical analysis

The chemical composition of each deposit layer (outer, inner and interface) of the pre-protector was analyzed by a comprehensive wet chemical analysis method which takes into account large area and different particle sizes as discussed later. The concentrations of these elements are presented in Table 1.

**Table 1:** Elemental analysis of deposits of pre-protector in terms of growth.

Position	Major element
Outer	Cl (7.82%), Ca (8.13%), Si (2.25%), Al (3.29%), S (10.5%)
Inner	Ca (6.32%), Cl (5.58%), Na (5.89%), S (10.01%), Si (4.35%), K (4.93%)
Interface	Na (7.54%), S (14.63%), K (6.15%), Cl (1.36%)

The elements detected in the interface layer are K, Cl, Na, and S which could be KCl, NaCl, and K_2_SO_4_. The co-existence of alkali chlorides and sulfates in the interface of the deposits results in the formation of eutectic compounds of low melting point^27^ which melt at low temperatures and cause corrosion by forming a flux with the protective oxide layer as well as providing a liquid phase for ionic charge transfer and for electrochemical attack, therefore, the interface layer plays a major role in accelerating corrosion as well as the medium for the exchange of ions between the metal substrate and the deposits. In addition, the eutectic low melting compounds of KCl-K_2_SO_4_ are sticky and can trap the coarse ash particles mainly containing silicon and calcium and this further promotes the development of the deposit. This is the reason why the inner layer contains a considerable amount of quartz and calcium sulfates (see Table 1). This observation is in good agreement with Niu *et al*.^28^. The holes accumulating together in the inner layer presumably were due to silica and calcium sulfate particles. These particles are slow to fuse or sinter in the deposit, so they contribute to both the granular/porous nature of the deposit^29^ and make the inner and the outer deposit layers permeable to the combustion gases to penetrate to the interface layer^30^, thus permitting the continuation of chlorination of transition metals at the interface.

**Figure 5:**
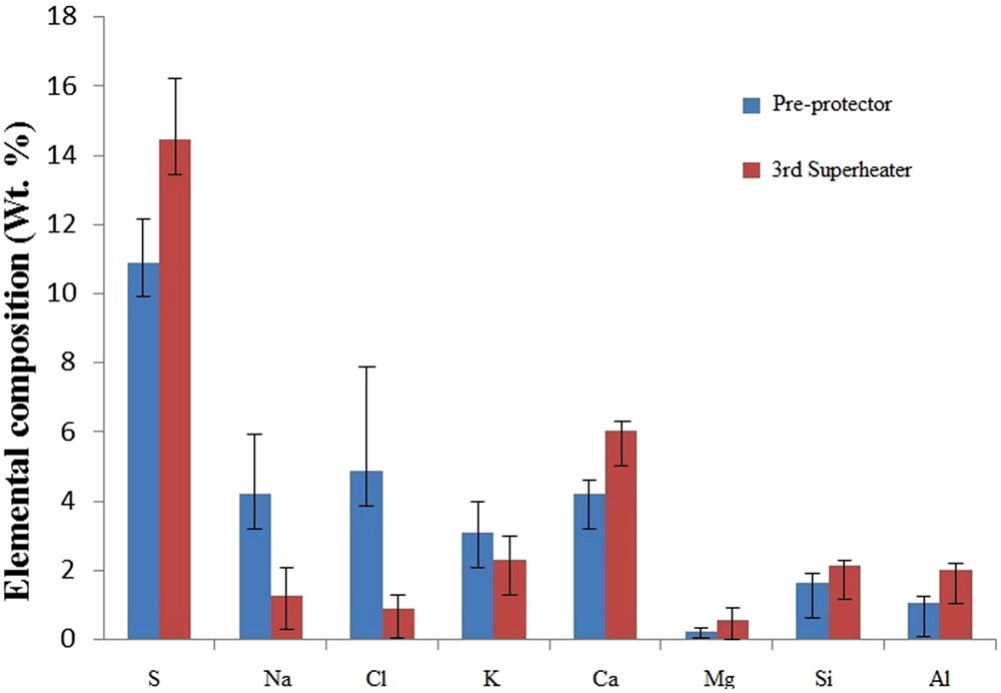
Elemental composition of deposits from pre-protector and 3^rd^ superheater by wet chemical analysis.

Since the sample of each layer was a mixed portion of three subsamples, the data was not used for the calculation of the overall deposit concentration because the standard deviation could not be computed from mixed samples. Therefore, in order to find the overall chemical composition of the whole deposit layer, resampling of individual samples were conducted. Five individual ash samples from randomly selected areas with varying depth from the outer surface to the interface were collected and subjected to wet chemical analysis and the results are presented in Fig. 5. The results show that the concentrations of Na and Cl were higher on the pre-protector than on the 3^rd^ superheater while the concentration of S and Ca were higher on the 3^rd^ superheater than the pre-protector. The error bars for S, Ca, Cl, and Na do not overlap; therefore, there is a significant difference in concentration between the pre-protector and the 3^rd^ superheater. The measurement uncertainties represented by error bars (standard deviation) on both locations were approximately similar, but the deviation between the average values and the standard deviation are much larger for Cl on the pre-protector. This observation can be attributed to the different composition and concentrations of the elements in particles in the deposits. At the surface, the deposits are dominated by irregularly shaped particles with more rod-shaped, which had a high content of chlorine and sodium while in the inner layer; particles rich in Si and S were dominant resulting in a considerable deviation for Cl.

During the flight of the flue gas from the combustion chamber to the convective section, flue gas temperature decreases and the volatile alkali chlorides condense. The vapor-condensation of alkali chlorides (NaCl and KCl) from the flue gas at 700 °C is higher than the flue gas at 550 °C^31^. Since the flue gas temperature at the pre-protector is around 700 °C, condensation of alkali chloride might have occurred at the pre-protector than at the 3^rd^ superheater where the temperature is around 500 °C. This might explain the occurrence of more amounts of Na and Cl on the pre-protector than on the 3^rd^ superheater.

### Comparison of the EDS analysis and wet chemical analysis

During the analysis of the deposits by EDS, it was observed that EDS had some shortcomings. The deposit had a depth profile consisting of coarse ash particles on the outer surface while smaller particles inside the particles and in the deposits. This led to a biased chemical composition due to the limited penetration depth of the electron beam. In addition, the EDS analysis is more sensitive to the surface composition of the larger particles than to the average bulk composition. It can also give strong deviations if particles comprise of a surrounding shell which is a property for condensation on solid nuclei^32^.

In contrast, the wet chemical analysis is a comprehensive technique that prevents all the flaws of the EDS investigation because all particles were considered with a good representation of their mass fractions, the bulk, and also takes into account the whole deposit thickness.

Although data in Figs 3 and 5 are in reasonable agreement, the concentration values of the elements are not the same. The average elemental concentrations from the wet chemical analysis (Fig. 5) are slightly less than those of the EDS analysis (Fig. 3). This was ascribed to the different concentrations of elements with varying depth which affected the average values from the wet chemical analysis. Furthermore, the amounts of large particles containing higher salt concentrations might have been underrepresented in the images investigated by the EDS. It was observed that performing EDS analysis in numerous areas over the entire deposit surface improved the agreement of elemental concentration with the wet chemical analysis method.

### XRD deposit analysis

**Figure 6:**
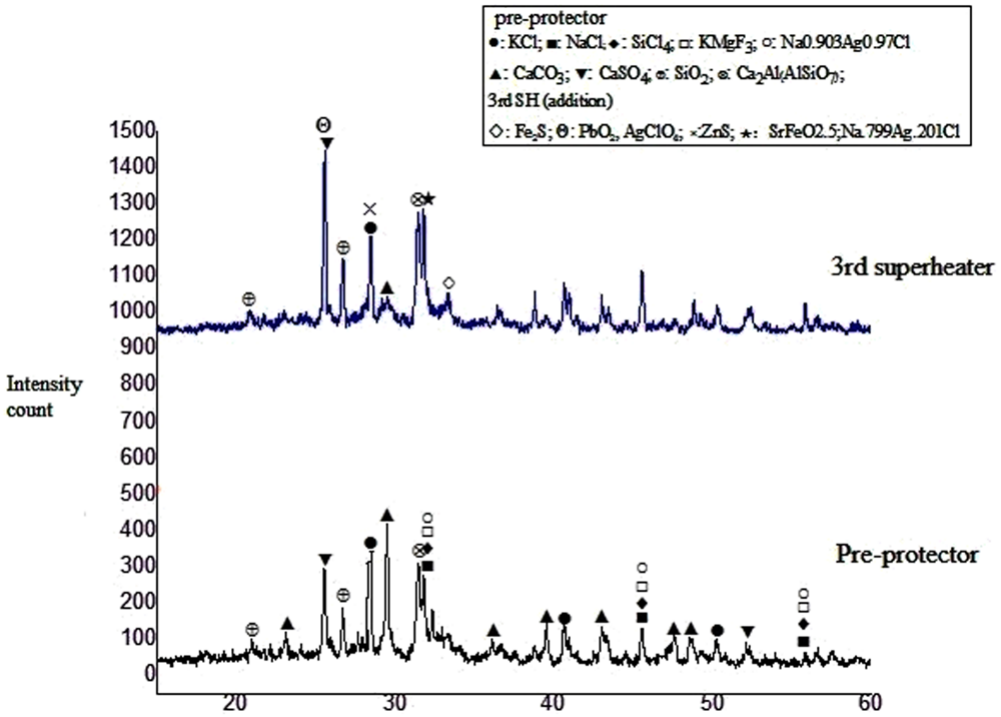
XRD results of deposits from 3^rd^ superheater and pre-protector.

Results from the X-ray diffraction (XRD) analysis of the deposits from the pre-protector and the 3^rd^ superheater are shown in Fig. 6. Deposits of the 3^rd^ superheater showed a large content of PbO and CaSO_4_. The observation of calcium sulfate is in contrast with^26,33^. The possible explanation might be due to the prolonged exposure of the unsulfated CaO in the deposits to the combustion flue gas which may have resulted in the formation of CaSO_4_. This explanation is supported by findings of Lai *et al*.^34^ and Tang *et al*.^24^ that deposit particles consist of coarse particles rich in O, Ca, S, and C which might be CaSO_4_ and calcite (CaCO_3_). Furthermore, CaO might have been formed from Ca metal at high oxygen partial pressure and low temperatures over a short period of time^32,35^.

Even though limited by the deposition selectivity, the deposits contained SiO_2_ which may have resulted from the sticking capture and inertial impaction. KCl was present in considerable amounts on the 3^rd^ superheater but was less than CaSO_4_. Various types of iron compounds such as Fe_2_S and SrFeO_2_ were also detected in the 3^rd^ superheater deposits. At pre-protector, the deposit was high in KCl and slight less CaSO_4_ as compared to the 3^rd^ superheater. The deposits also contained sorosilicate gehlenite (Ca_2_Al(AlSiO_7_)) which possibly comes from the solid-state reaction of CaO with Al_2_O_3_ and SiO_2_ at high temperatures^36^. The deposits on the pre-protector contained large content of calcium carbonate, calcium sulfate, silicon tetrachloride as well as alkali chlorides (KCl and NaCl). Some diffraction peaks were left unidentified, implying that other compounds were probably present in the examined deposits.

### Corrosion observation

**Figure 7:**
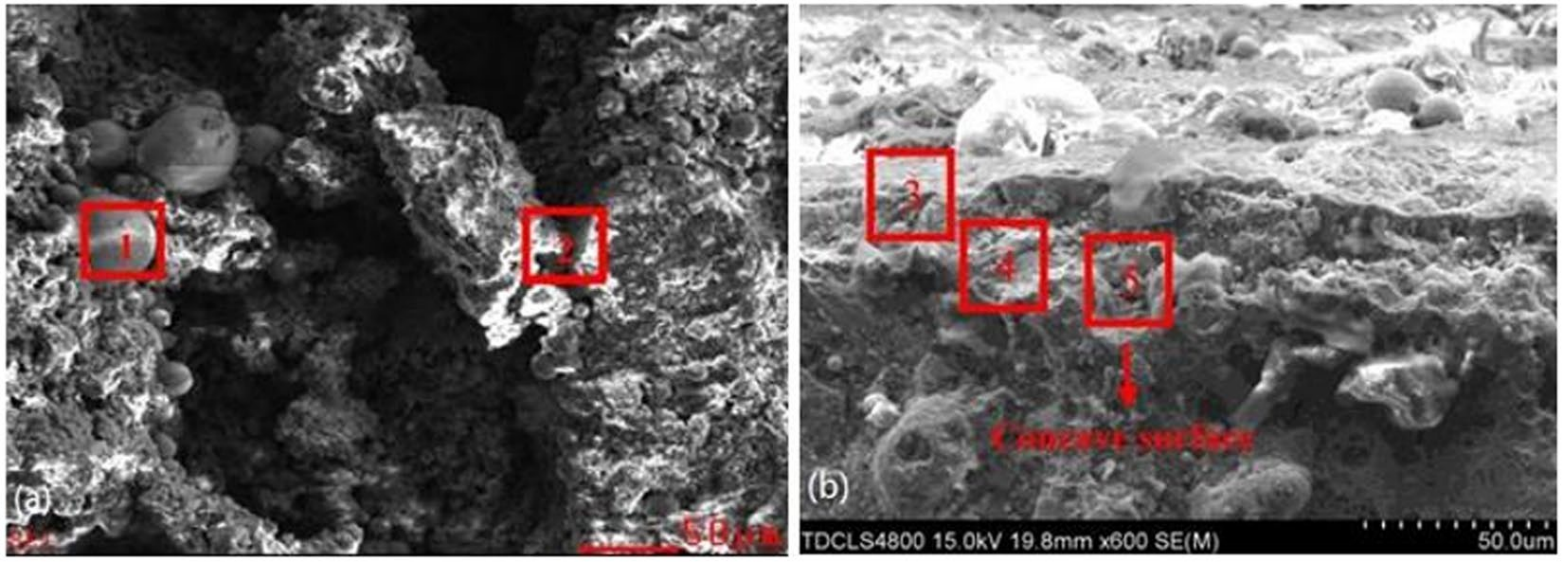
SEM micrograph of (**a**) pre-protector’s surface and (**b**) cross-sectional area.

The morphology of the corroded tube’s surface and cross-sectional area for the pre-protector are displayed in Fig. 7. There are many particles of various shapes scattered on the corroded surface of the pre-protector: a spherical-shaped particle (1#) and a rod-shaped particle (2#). From the EDS analysis, it was observed that the spherical-shaped particle is the transition metal chloride. However, only a few spherical-shaped particles were found and most of them were surrounded by the rod-shaped particles, which had a high content of chlorine, sodium, and lead. Particle (2#) was identified as the outer layer of the deposits in terms of structure. This means severe chlorine-induced deposition and further corrosion occurred on the surface of the pre-protector. Therefore, the cross-sectional area of the pre-protector was analyzed vertically and marked as Gap (3#, 4#, and 5#) in Fig. 7(b). It was found that there was a decrease of chlorine content from the outer surface (Gap 3#) to the interface (Gap 5#). This was probably due to the vaporization of iron chloride from the interface to the outer layer during the active oxidation cycle.

**Figure 8:**
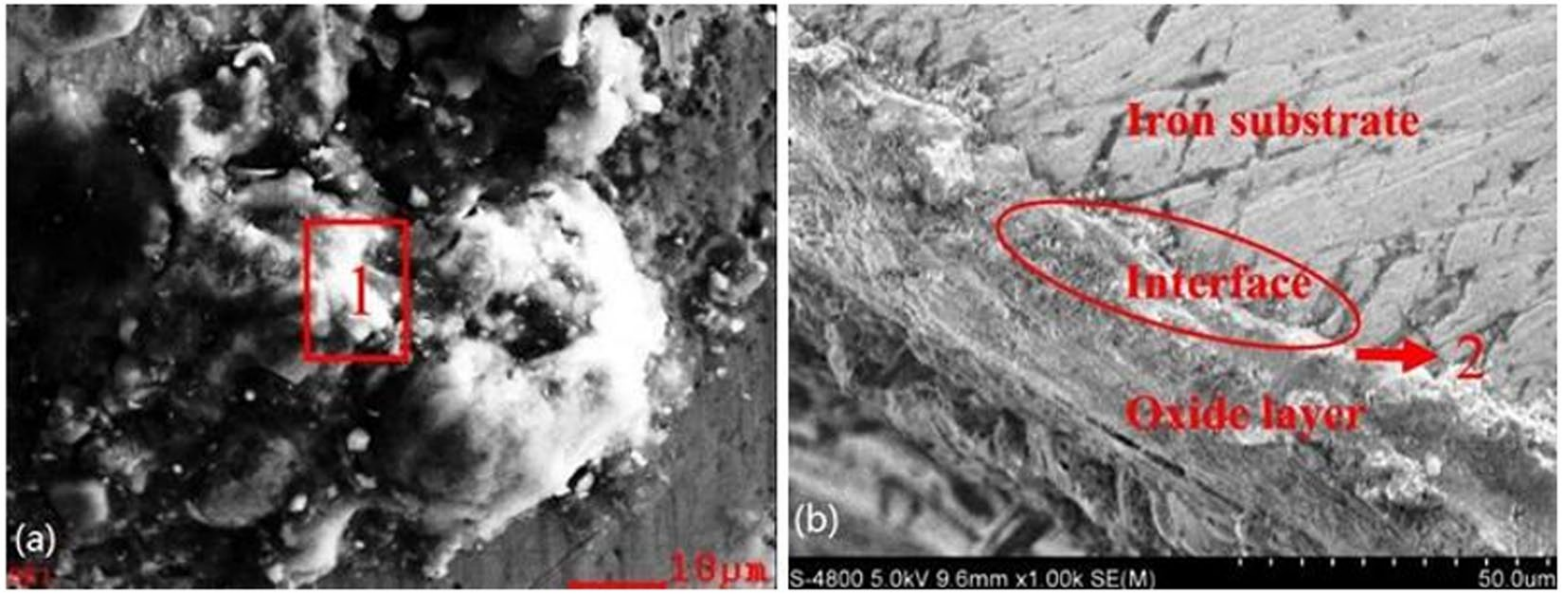
SEM micrograph of (**a**) 3^rd^ superheater’s surface and (**b**) cross-sectional area.

The slagging surface and cross-section of the 3^rd^ superheater are shown in Fig. 8. From Fig. 8(a), Gap (1#), the massive aggregation of ash deposits was selected and chemically analyzed. The elemental composition of Gap (1#) consisted of a high content of sulfur, sodium, and potassium with lack of calcium and magnesium. In Fig. 8(b), it was visible that the delamination of metal and oxide layer was obvious and the metal/oxide interface was marked as Gap (2#). Some scratches were found scattered in the interface and iron substance. In addition, the chemical composition of Gap (2#) showed that the content of chlorine at the 3^rd^ superheater was slightly less than that at the pre-protector (Gap (5#) in Fig. 7), which proved that the degree of corrosion on the 3^rd^ superheater was less than the pre-protector.

## Discussion

The purpose of this study was to investigate the deposit chemistry mainly deposit morphology and concentration of corrosive elements at the pre-protector and the 3^rd^ superheater as well as evaluating their corrosion tendency. The results show that the deposit from both locations had diverse microstructural particles including spherical, elongated, plate-shaped and hemispherical. This observation is consistent with^25^. Spherical particles could have resulted from the solidification of liquid droplets during cool-down and the other shaped particles might presumably have been emitted from the furnace as solid particles and may have a higher melting point than the spherical particles due to a different chemical composition^25^. The diversity in the microstructure of the particles was due to the different deposits minerals shown in Fig. 6, which had resulted from the physicochemical reactions that took place during the deposit formation and growth.

The deposit on the 3^rd^ superheater had a large amount of Ca and S while the pre-protector was high in Na, K, Cl, and S indicating the presence of CaSO_4_ and a eutectic salt of alkali chloride and sulfate respectively. A large amount of sulfur in deposits was due to the sulfation of alkali chlorides at high steam temperature forming thin and dense alkali sulfates layer at the metal/oxide interface^37^. Different from the role of calcium sulfate, alkali chlorides especially those in the deposits are more severe than the other species like HCl in the flue gas, due to the formation of eutectic low-melting compounds^27^. These compounds melt at low temperatures and cause corrosion by forming a flux with the protective oxide layer as well as providing a liquid phase for ionic charge transfer for electrochemical attack^5^.

The detection of more sulfur at the interface than in the outer layer is in strong agreement with literature^20,38^ and it is assumed to be due to sulfation of alkali chloride by SO_3_ which might have been catalytically formed within the deposit by the Fe_2_O_3_ in the oxide layer^39^. Moreover, it is assumed that SO_2_ might have diffused through the micro-cracks of the oxide layer and reacted with alkali chlorides, which were primarily deposited near the interface forming alkali sulfates^40^.

The pre-protector, which is arranged prior to the 3^rd^ superheater in waste boilers, has higher flue gas temperature (650 °C) leading to an increased surface temperature. The amount of alkali and chlorine in the deposits is high, while that of Ca is lower as shown in Figs 3 and 5. Other ash-forming elements, including aluminum, magnesium, and silicon occurred in minor quantities at all points, but the main elements differed between points. A study by Baxter^41^ showed that the deposition mechanisms include condensation, inertial impaction, thermophoresis, eddy impaction and chemical reaction. Liu *et al*.^42^ explained using Johnson-Kendall-Roberts theory that for a general deposition of a particle, a certain critical velocity should be achieved in order for the particle to either rebound or deposit. The particle diameter negatively influences its critical velocity, implying that smaller particles adhere easily to the surface of the boilers^42^. Since the pre-protector has a temperature around 700 °C, the bonding energy between the surface and particle is in this range. This indicates that during the early stage of deposition, thermophoretic and condensation mechanisms of sub-micrometer particles are dominant. When the deposits increase in mass, ash heat-transfer coefficient decreases, resulting in an increase in the ash deposit surface temperature, and subsequently lead to the partial melting of ash deposits at the top layer. For example, K_2_SO_4_ has a melting point 1069 °C but decreases to 694 °C when reacted with potassium chloride to form eutectic KCl-K_2_SO_4_^5^. The deposit which is partially melted becomes more adhesive; hence accelerate the impaction of particles. That is why the pre-protector ash deposit formed around 700 °C had high amounts of alkali, S, and Cl. At the 3^rd^ superheater, which is arranged after the pre-protector where the deposition temperature is around 400 °C, a large temperature gradient between the metal temperature and the flue gas exists. This large temperature difference promotes thermophoretic/condensation mechanisms of fine particles. According to Tang *et al*.^24^, the ultrafine sub-micrometer particles act like “glue” during the ash deposition at lower temperatures. CaO are fine particles and have a large specific surface area, therefore they influence their critical velocity resulting in the large adhesion force to the metal surface which leads to the aggregation of fine CaO particles on heated surfaces, forming ash deposits^43^. The precipitation and accumulation of fine CaO particles on the 3^rd^ superheater are the reason why more CaSO_4_ as binders or as particles were detected in the deposits. CaO and or CaCO_3_ acting as a paste between the particles result in the formation of CaSO_4_. Sulfates, however, are less corrosive and only play the main role in preventing penetration of gases to the interface^39^.

The chemical composition of the ash deposits was important to the reveal of the deposition mechanism. SEM analysis shows that the pre-protector deposits consist of thicker and denser particles while the 3^rd^ superheater deposits consist of thinner and fine particles. Based on the composition and morphology of the deposits investigated, this study has taken a step in the understanding of deposit formation at the pre-protector and the 3^rd^ superheater. Chemical composition analysis showed that deposits at the 3^rd^ superheater had high Ca and S compounds, in which calcium-rich compounds in the deposits went through sulfation process. Xu *et al*.^44^ mentioned that the ultrafine sub-micrometer particles act as adhesive agents during deposit formation at low temperatures. Ca compounds (CaO and CaCO_3_) released from the MSW fuel, are ultrafine particles and have a large specific surface area, which leads to a large binding force among them. These particles aggregate on the 3^rd^ superheater surfaces through thermophoretic and condensation mechanisms due to a large temperature difference between the metal temperature and the flue gas^43^. Moreover, condensed elements on this layer result in the formation of a sticky deposit layer in which fly ash particles bond through impaction process. Sulfates largely condense at high temperatures than chlorides and are more thermodynamically favored as temperatures decrease, delaying volatilization at lower temperatures, thus detected in more concentration at the 3^rd^ superheater where the metal temperature is relatively low. KCl-K_2_SO_4_ is a low melting compound formed from the reaction of KCl and K_2_SO_4_ in which the later was formed from sulfation of alkali chloride making the deposit more sticky for capturing of fly ash particles^45^.

At the pre-protector, the chemical composition detected suggests a different mechanism. The high temperature of deposit layer makes it easily sintered, and therefore increases the probability of ash particles to bond themselves through inertial impaction^24^. The alkali metals released in the flue gas as aerosols of sulfates, chlorides, and hydroxide go through mineralogical transformations and chemical reactions^24^. These aerosols then undergo nucleation, adsorption, condensation thereby grows to form submicrometer ash particles as the flue gas temperature decreases and then stick on the heating surfaces forming an adhesive deposit layer through thermophoresis and turbulent diffusion. Some mineralogical reactions between sulfates, chlorides and the flue gas took place, among which anhydrite calcium phase (CaSO_4_) as a matrix likes binding material between the grains was formed^16,20^. Fly ash particles adhesively bond themselves onto this layer through inertial impaction^21^.

According to Otsuka^31^, the molecular quantity of vapor-condensed NaCl and KCl increased with increasing flue gas temperature between 550–800 °C. This is because KCl and NaCl do not volatilize at lower temperatures due to high binding energy (NaCl 787 kJ/mol, KCl 717 kJ/mol)^46^. As the flue gas temperature continues to increase to ~800 °C, alkali chlorides (NaCl and KCl) volatilize forming a gas rich in chlorine compounds^46^. Therefore, flue gas temperature around pre-protector certainly led to the increase of chlorides on tube deposits, which confirmed that deposits at the pre-protector contained more alkali and chlorine with less Ca and S as shown in Figs 3 and 5. Furthermore, from the corrosion point of view, more chlorine was detected in the deposits of the pre-protector than the 3^rd^ superheater, which proved that pre-protector suffered from severe corrosion as seen in Fig. 7. Notwithstanding the limitations of measuring the corrosion rate, this study suggests that the pre-protector deposits with Cl content ~4.86wt.% corroded the steel more than the 3^rd^ superheater deposits with less Cl ~0.9wt.%. This result is consistent with^47^. They observed that corrosion by superheater tube-deposits was less due to the stability of superheater tube-deposits, where the fusion of salt constituents in the deposits could not take place when heated at 400 °C. The superheater tube-deposits with chlorine <2wt.% is likely to remain solid at 400 °C, and therefore corrosion is reduced^47^. Since chlorine content found in the present study at 3^rd^ superheater where the metal temperature is normally 400 °C is less than 2wt.%, the salts are likely to remain solid reducing corrosion than the pre-protector with higher chlorine content and higher temperature. Moreover, the presences of high sulfur content reduce corrosion by sulfation of alkali chlorides^37,48^. This might presumably be the reason why the 3^rd^ superheater experienced a lower corrosion than the pre-protector.

As deposition is the driven reason for corrosion, an effective physical way using a weight-driven device for deposit removal to reduce corrosion can be applied in this WIP. The principle of this device is utilizing a small capacity motor as the power to drive a long axis at a low speed. There are many vibration hammers hanging in the long axis beating the heat transfer surface in boilers to let the ash deposit fall off from the tubes. The advantage of mechanical vibration and beating is less power consumption and no additions to the flue gas. But this would not be good for the weld seam strength of the boiler tubes and might lead to the reduction of its service life and also enhanced corrosion-erosion.

Chemical way to reduce chlorine-induced corrosion can be employed by adding sulfate compounds to convert alkali chlorides into less corrosive alkali sulfates and HCl. Vattenfall Company, one of the European’s largest generators of electricity and heat, adopts adding ammonium sulfate to waste boilers by the real-time monitoring system. This was already used in a fluidized bed boiler firing biomass in Poland^49^. Moreover, optimizing the combustion environment by reducing the temperature can prevent the deposition of alkali chlorides on the pre-protector while facilitating the formation of calcium sulfates which are less corrosive. Although this approach can reduce the boiler efficiency, the service life of the boiler can be prolonged and reduce the maintenance cost.

## Experimental

### Materials

Waste samples collected from the Chengdu waste incineration plant were sorted into single fractions and then categorized into three groups: organic, inorganic, and recyclable wastes in order to obtain a better understanding of the waste composition. The organic part consisted mainly of food wastes ~43.63wt.% and grass ~0.99wt.%; inorganic wastes comprised of ashes ~10.91wt.% and bricks ~0.24wt.%; and recyclable part was composed mainly of plastics ~23.52wt.%, papers ~13.12wt.%, textiles ~3.67wt.%, glasses ~3.36 wt.% and metals ~0.55wt.%. The waste samples were tested using an elemental analyzer (Analytik Jena multi ® EA 2000) for the ultimate analysis shown in Table 2 following the American Standards for Testing and Materials D5373–2008 criterion. The organic part, especially the food residues occupied approximately half of the waste composition which is similar to other Chinese cities^50^. Also, plastics in recyclable part take a large proportion which occupied almost a quarter of the waste composition. Plastics and food residues have been reported as the main source of chlorine (~0.5–1.0wt.%)^11^, therefore chlorine content was tested following the method described elsewhere^11^, and thus a chlorine concentration of 1.09% was found (Table 2). The composition of MSW and ultimate analysis were the average values obtained in winter in 2015. Sampling and analysis methods for MSW fuels were based on CJ/T 313–2009 criterion^51^.

**Table 2:** Proximate analysis and ultimate analysis of waste in Chengdu (wt.%).

Proximate analysis (wt.%)			LHV (kJ/kg)	Ultimate analysis (wt.%)					
Moisture	Volatile^*^	Ash		C	H	O^*^	N	S	Cl
41.76	42.19	16.05	6280	42.38	7.17	47.16	2.13	0.07	1.09

^*^By difference.

LHV-Lower heating value.

### Plant introduction

Experiments were conducted in a WIP in Chengdu, China. This plant consists of three boilers with the total capacity of 1800 tons per day of MSW collected from three districts in Chengdu. The energy recovery system is designed for power generation and heat net. The Unit I boiler was selected as the tested boiler and its operational parameters are shown in Table 3.

**Table 3:** Specifications and operating parameters of the Unit 1 boiler.

Nominal capacity	Nominal electricity production	Primary air flow	Secondary air flow	Steam temperature	Steam pressure
600 Tons/day	12 MWe	30~35 × 10^3^ Nm^3^/h	6~8 × 10^3^ Nm^3^/h	400 °C	4 MPa

**Figure 9:**
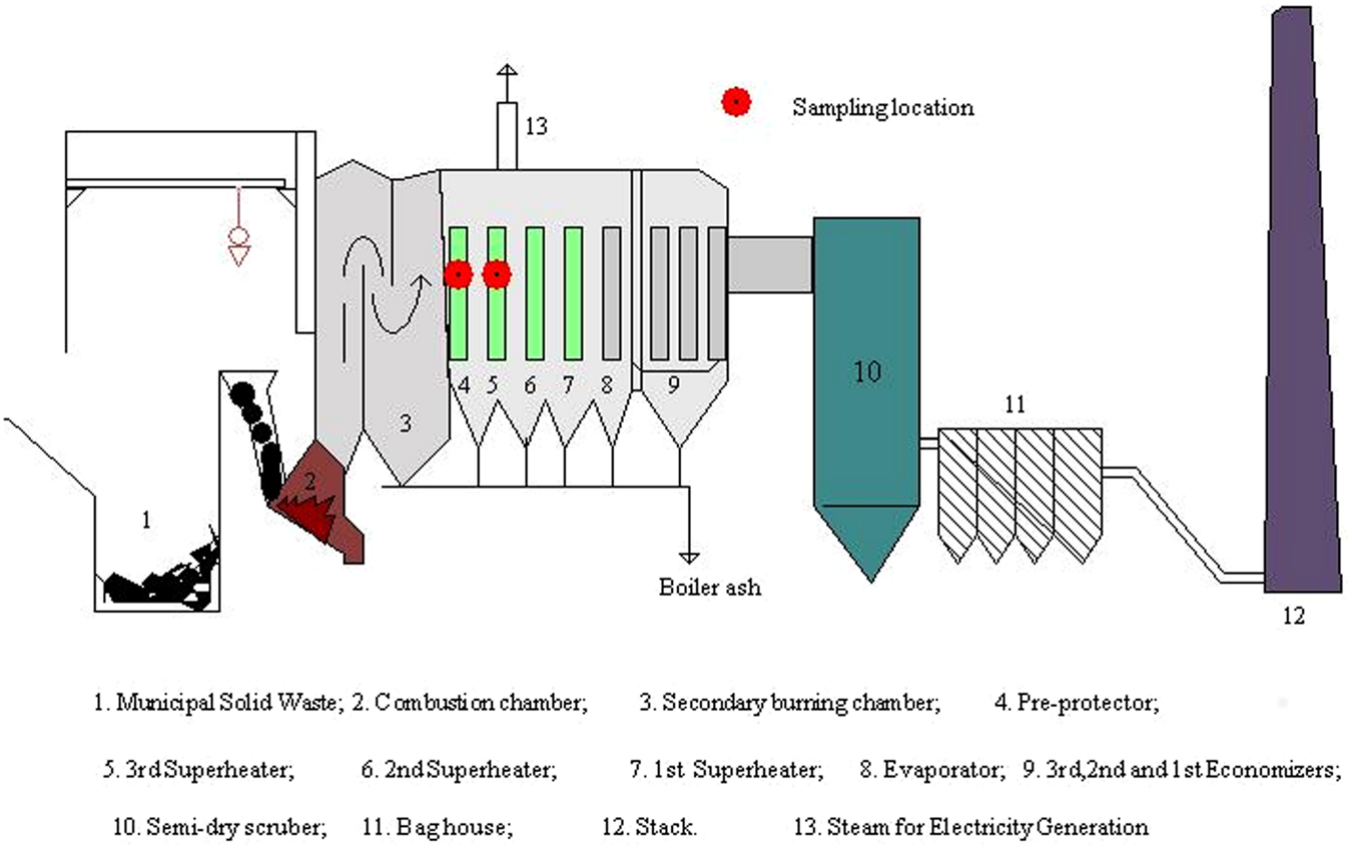
Schematic diagram of the WIP in Chengdu.

The schematic diagram of this WIP is shown in Fig. 9. During the experiments, there were stable operations and the boiler did not experience any malfunctions. The flue gas passed through the furnace chamber, the secondary burning chamber in the second pass of the boiler, the pre-protector, the tertiary superheater (3^rd^ SH), the secondary superheater (2^nd^ SH), and the primary superheater (1^st^ SH). After primary superheater, the gas passed through the evaporator and three economizers (3^rd^ EC, 2^nd^ EC, and 1st EC). Finally, the fly ash particles were removed in a semi-dry scrubber. Several thermocouples were set near the probes to monitor the flue gas temperature. The steel material used for the pre-protector was 20 G (F-C) and is composed of 0.21% C, 0.5% Mn, 0.27% Si, 0.03% S, 0.03% P, 0.3% Cr and Fe as balance. The ring probes of 20 G samples were cut into 50 mm in length and ground with 800# grit SiC paper. The probes were cleaned and degreased with acetone in an ultrasonic bath and subsequently dried at 105 °C until no weight change. Probes were then set at the pre-protector and the 3^rd^ superheater.

### Deposit sampling

After one-year exposure, ash deposit samples were collected from the pre-protector as well as the 3^rd^ superheater and were subsequently stored in airtight containers to exclude atmospheric moisture as some products are hygroscopic. Five samples were collected from the pre-protector and two samples from the 3^rd^ superheater. The ash deposit samples were removed from the tubes in different portions and depth with a knife in order to find the overall concentrations of K, Na, Cl, S, and Ca in the deposit. The other elements analyzed are shown in the Supplementary information (Supplementary Table S1). In order to determine the chemical composition in terms of the deposit growth on pre-protector, three ash samples of each layer were collected from different locations and mixed forming a composite sample for the outer layer, inner layer, and interface. The deposit sampling was done by collecting ash deposits for the outer layer to a depth of 3 mm on three separate positions, mix them and subject it to wet chemical analysis; then scrape the deposit to the middle layer to collect ash deposits and finally scrape until close to metal to collect samples for the interface. This procedure was done as it gives more accurate amounts of elements than simple EDS, which analyses on small portions. Thereafter, the probes were carefully removed from the pre-protector and the 3^rd^ superheater. They were cut into four pieces and the cross sections were polished and dried for further analyses while preserving the integrity of the deposits in order to investigate the corrosion attack.

### Ash deposits analysis

Wet chemical methods were used to analyze the deposits and were done on the windward side of the ring. To determine the concentration of Cl, we have detailed well the procedure in our previous paper^11^. In the chemical analyses of deposits for calcium concentrations, Na_2_B_4_O_7_ was fluxed on the deposits and then analyzed using ICP-AES. Na and K contents were determined by adding lithium borate to the deposits and then analyzed using Atomic Absorption Spectroscopy. Sulfur contents were obtained by iodine titration. These procedures were done to determine the actual quantities of K, Cl, Na, Ca, and S coming from the flue gas that was deposited on the pre-protector and the 3^rd^ superheater and also determines their concentrations in terms of deposit growth on pre-protector.

In addition, the morphology of the deposit surface was analyzed using scanning electron microscopy (SEM) (Philips XL-30 TMP ESEM, 20 kV, Holland). The images obtained were supplemented by EDS analysis for comparison with wet chemical methods. Furthermore, the ash deposits from both locations were ground, passed through a sieve with a mesh size of 70 mm and were stored in desiccators. X-ray Diffraction (XRD) study was then conducted to determine the crystalline compositions in the deposits using a Phillips X-pert X-ray diffraction system with CuK*α* radiation.

### Data availability

All data generated and analyzed during this study are included in this published article. Raw wet chemical analysis data used to plot Fig. 5 are provided in Supplementary Table S1.

## Conclusion

Municipal solid waste incineration results in severe deposition and corrosion in WIP. Troublesome elements to these problems include K, Na, Cl, Ca, and S. A comparative investigation of deposit chemistry and corrosion of the pre-protector and the 3^rd^ superheater at a 36 MWe WIP showed that; the deposits from the pre-protector are rich in Cl, K, and Na while deposit from the 3^rd^ superheater contains more Ca and S. The concentration of alkali decreased from the interface to the outer layer while Cl increased in the same direction in deposits at the pre-protector. The concentration of Ca and S increased from the outer layer of the deposits to the interface while alkali decreased in the same trend in the deposits at the 3^rd^ superheater. The deposit formation at the pre-protector was mainly influenced by alkali chlorides while at the 3^rd^ superheater by ultrafine sub-micrometer Ca-rich compounds. Based on the deposits element composition and corrosion of the metal samples, we conclude that pre-protector ash deposits are more severe than the 3^rd^ superheater ash deposits. However, the interaction of alkali metals and chlorine in corrosion warrant further research.

## Electronic supplementary material

Supplementary Information
