# Supplementary material for: Full-scale experimental investigation of deposition and corrosion of pre-protector and 3rd superheater in a waste incineration plant
Source: Sci Rep. 2017 Dec 13;7:17549. doi: 10.1038/s41598-017-17438-3 (PMC5727521; doi:10.1038/s41598-017-17438-3)
Supplement: Supplementary file 1 — Supplementary Information [file 41598_2017_17438_MOESM1_ESM.pdf]

Full-scale experimental investigation of deposition and corrosion of pre-protector and 3rd superheater in a waste incineration plant

Wenchao Ma, Wenga Terrence, Nan Zhang, Guanyi Chen, Beibei Yan, Zhihua Zhou, Xiao Wu

Supplementary Table S1

Wet chemical analysis on five samples of the deposits collected at pre-protector and two samples from the deposits of the 3rd superheater.

Position		C	O	Na	S	Mg	Al	Si	Cl	K	Ca	Fe	Pb
Pre-protector	1	30.7	8.99	4.57	10.17	--	2.12	1.37	3.42	2.34	5.02	53.34	--
	2	23.35	10.49	3.89	9.32	--	0.92	1.82	11.53	2.72	4.4	28.6	19.2
	3	31.23	30.41	3.28	10.73	0.17	1.84	--	3.84	2.63	3.9	34.23	--
	4	32.72	19.66	2.36	--	0.33	--	--	4.28	3.73	--	44.01	--
	5	42.31	18.66	6.98	12.3	--	--	--	1.23	4.44	--	35.42	--
Mean value		32.06	17.64	4.22	10.63	0.25	1.38	1.61	4.86	3.17	4.71	39.12	19.2
3rd SH	1	32.57	31.94	0.72	15.7	0.40	2.02	1.98	1.18	1.87	5.8	30.23	--
	2	25.95	15.32	1.84	13.2	0.75	2.28	2.3	0.61	2.82	6.29	45.13	--
Mean value		29.26	23.63	1.28	14.45	0.58	2.15	2.14	0.9	2.35	6.05	37.68	--
